# Modes Coupling Analysis of Surface Plasmon Polaritons Based Resonance Manipulation in Infrared Metamaterial Absorber

**DOI:** 10.1038/srep46093

**Published:** 2017-04-11

**Authors:** Guoshuai Zhen, Peiheng Zhou, Xiaojia Luo, Jianliang Xie, Longjiang Deng

**Affiliations:** 1 National Engineering Research Center of Electromagnetic Radiation Control Materials, State Key Laboratory of Electronic Thin Film and Integrated Devices, University of Electronic Science and Technology of China, Chengdu 610054, Sichuan, People’s Republic of China

## Abstract

Surface plasmon polaritons (SPPs) and standing wave modes provide interesting and exotic properties for infrared metamaterial absorbers. Coupling of these modes promises further development in this field but restricted by the complexity of modes analysis. In this work, we investigate the general phenomenon of modes coupling supported by a metal (with grating)-dielectric-metal sandwich structure based on rigorous coupled-wave analysis (RCWA) method and experiment results. Through the analysis of fundamental modes, a new approach based on the boundary conditions is introduced to reveal the coupling mechanism and the corresponding resonance shifting phenomenon with simple but rigorous derivations. The strong coupling between SPPs excited on the dielectric-metal interfaces and rigorous modes of standing waves in the dielectric layer can be manipulated to improve the detection sensitivity of sensors and emissivity efficiency of infrared emitters.

Surface plasmon polaritons (SPPs) can greatly enhance local electromagnetic field and tightly confine the enhanced electromagnetic energy to the metal-dielectric surface^1^,^2^, which promises broad application prospects, such as high performance transmission^3^, near-field imaging^4^, sensing^5^, solar cell^6^, and so on. Since the concept of SPPs was proposed, increasing researches have been carried out to explore how to excite and manipulate SPPs efficiently. Recently, resonant metamaterial absorbers featured by metal-dielectric-metal sandwich structures, have become one of the main resources to manipulate SPPs with different periodic patterns such as one-dimensional (1D) grating^7^,^8^,^9^,^10^, 2D disk arrays^11^,^12^, patch arrays^13^, cross bars^14^ and aperiodic patterns such as nanoparticles^15^ and single cavity^16^. The interactions between SPPs and other kinds of resonances, e.g. magnetic polaritons (MP)^17^,^18^, cavity resonance^19^, and Fabry-Perot resonance^20^, have brought exotic characteristics in infrared spectral absorption^21^,^22^.

Even though many researches have been developed in this field, most of the sandwich structures possessed a very thin dielectric spacing layer, typically lower than one tenth of the incident wavelength^23^,^24^,^25^. In this condition, the reported SPPs are mainly excited on the air-metal surface and only a few of them are excited on the dielectric-metal interface^26^. As for the sandwich structure absorbers decorated by surface patterns, once SPPs on the dielectric-metal surface is excited, SPPs on the upper and lower dielectric-metal interfaces may be coupled and further complicated by the discontinuity of the upper interface patterns. On the other hand, standing waves have been introduced to explain the electromagnetic scattering problems of the metal-insulator-metal infrared absorber^16^,^27^,^28^. In this paper, we focus on standing waves between the upper metallic grating and the lower metallic base. When the thickness of dielectric spacing layer is comparable to the incident wavelength, this kind of standing waves will have a significant effect on electromagnetic absorption and they may be coupled with SPPs on the dielectric-metal interfaces and generate new absorption phenomenon. In addition, as both standing waves and SPPs could be expressed in the form of plane waves, their existence can be judged directly and effectively by the distribution of the Floquet modes^29^,^30^,^31^ but fewer researches have tried in this way.

To reveal the modes excitation and coupling phenomenon in SPPs structures, a sandwich structure consists of one-dimensional metal grating and relatively thick dielectric spacing layer is proposed, fabricated and measured for infrared absorption in this paper. Simulation and experiment results of the infrared absorption spectrum are shown at first and then discussed by RCWA method to identify the absorption modes. In the “Modes Coupling Analysis” part, effective medium theory is employed to explain the shifting of SPPs excitation wavelength and a new approach based on the boundary conditions is introduced to reveal the coupling mechanism and the corresponding resonance shifting of standing wave line.

## Results and Discussion

### Modes Simulation and Experiments

**Figure 1:**
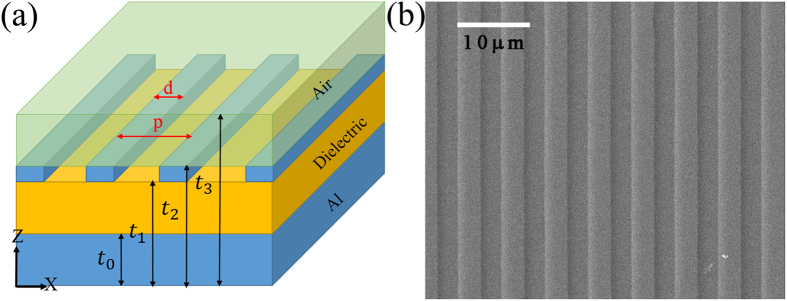
Configurations of the sandwich structured metamaterial absorber. (**a**) Schematic of the simulation model marked by four lateral positions (*t*_0_ to *t*_3_) to present the feature sections: Al, dielectric, Al grating and air. (**b**) Scanning electron microscopy (SEM) image of the fabricated sample. The Al grating has a period *p* = 6 μm and width *d* = 3 μm. The thickness of four lateral sections are *t*_0_ = 0.2 μm, *t*_1_ − *t*_0_ ranging from 0.05 to 8 μm, *t*_2_ − *t*_1_ = 0.1 μm and *t*_3_ − *t*_2_ = 2 μm.

Figure 1(a) illustrates the geometry of the sandwich structured metamaterial absorber for numerical simulation. Thickness of the bottom continuous and top grating Al layers are fixed, but the thickness of dielectric layer is varied. The structure is surrounded by air. Complex permittivity of Al is described by the Drude model with a plasma frequency of *ω*_*p*_ = 2π × 3.57 × 10^15^ rad/s and a collision frequency of *ω*_*c*_ = 2π × 19.41 × 10^12^ rad/s^32^. The dielectric loss and dispersion will make the coupling between SPPs and standing waves more complicated and the analysis with lossy dielectric is based on the analysis with lossless dielectric, so the relative permittivity of dielectric layer is intrinsically set to 2.28, employing lossless and non-dispersive dielectric parameter to illustrate our theory. To compare with experimental results, lossy and dispersive Al_2_O_3_ layer^33^ is further introduced for simulation. More details about the effects of dielectric loss and dispersion are shown in the Supporting Information. Scanning electron microscopy(SEM) images of the proposed metamaterial absorber prepared by standard e-beam deposition and UV lithography techniques is shown in Fig. 1(b), in accordance with Fig. 1(a). The 200 nm Al ground plane was first evaporated onto a silicon substrate and then covered by the Al_2_O_3_ layer with different thickness. Lamellar grating of the 100 nm Al film was realized by the lift-off lithography process on Al_2_O_3_ layer.

**Figure 2:**
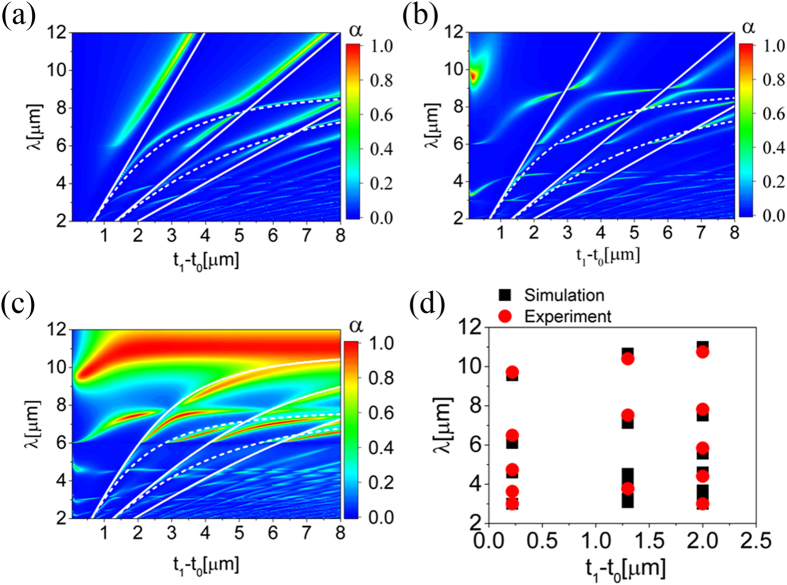
Simulation and experimental results of the sandwich structured metamaterial absorber at normal incidence. (**a**,**b**) Contour plots of the spectral absorption *α* as a function of incident wavelength *λ* and thickness of the lossless dielectric layer for TE (a) and TM (b) polarization. The white solid lines represent the predicted excitation of the zero order standing wave, while the dashed lines are for the first order one. (**c**) Contour plots of the spectral absorption *α* with Al_2_O_3_ layer for TM polarization. (**d**) Comparison of the absorption peak wavelengths between the simulation and experimental results. Three absorber samples selected at the characteristic regions of (**c**) are defined by the thickness of the Al_2_O_3_ layer, with *t*_1_ − *t*_0_ = 0.22 μm, 1.3 μm and 2 μm.

In simulation, a plane wave with wave vector 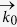 is incident at our structure from air. The RCWA method^34^,^35^ is employed for modes simulation by retrieving the amplitude and phase of each Floquet modes. In the case of TM polarization, the incident electric field is along the *X* direction and the total electromagnetic fields at any arbitrary point 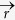 takes the form^35^


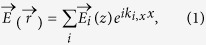



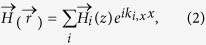



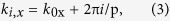


where *i* represents the order of the Floquet modes, *k*_0,*x*_ represents the *X*-component of the incident wave vector, and *p* represents the period in Fig. 1(a). In the rest of this paper, the *i*th order Floquet modes is written as *TM*_*i*_ for TM polarization and *TE*_*i*_ for TE polarization (with the incident electric field along the *Y* direction). Simulation results for the spectral absorption of the proposed absorber at normal incidence for both TM and TE polarization and the corresponding experimental results for TM polarization are shown in Fig. 2.

For TE polarization in Fig. 2(a), there is no SPPs but the excitation of standing waves. The strong absorption positions are along the standing waves lines or at the cross region where two different modes of standing waves couple together, especially when the incident wavelength is larger than 6 μm. This is an expected absorption pattern. In contrast, the absorption pattern in Fig. 2(b) is distinguished by two main features. One is the excitation of strong magnetic polaritons at the top-left corner, as our former works have shown^17^,^18^. The other is the twisting of absorption bands nearby the SPPs wavelengths of *λ* = 4.5 μm, 6 μm and 9 μm, which is the focus of this paper. Our theory is also successfully extended to lossy and dispersive dielectric in Fig. 2(c), which are the simulation results with Al_2_O_3_ the dielectric layer. Even though the relative permittivity of Al_2_O_3_ is strong dispersive in the 8–12 μm wavelength range^33^, it keeps a similar absorption pattern. The calculated excitation wavelengths of SPPs are *λ* = 4.62 μm, *λ* = 6 μm and *λ* = 7.83 μm, matching with the simulation results in Fig. 2(c). Detailed calculation can be found in the Supporting Information. In Fig. 2(d), three groups of the experimentally tested absorption peak wavelengths match with the simulation results in general. Existence of the proposed two features in the absorption spectrum of our absorber for TM polarization is therefore experimentally supported. More detailed experimental datas are provided in the Supporting Information.

### Identification of Absorption Modes

Since the Al_2_O_3_ layer is continuous and sandwiched by two metal layers, there will be standing waves between the upper Al grating and lower Al base. Considering the effects of Al base on tangential and normal electric field, the excitation condition of standing waves at normal incidence can be written as


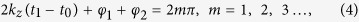


where *k*_*z*_ is the effective wave vector of the given Floquet mode in the dielectric layer, *t*_1_ − *t*_0_ represents the thickness of dielectric layer and varies, *φ*_*1*_and *φ*_*2*_ are the phase retardations on the upper and lower dielectric-metal interfaces, respectively. For the simplicity of analysis, the lossy metal is treated as perfect electric conductor (PEC) to employ the rigorous PEC boundary conditions, so *φ*_1_ = *φ*_2_ = π for the tangential electric field *E*_x_, and *φ*_1_ = *φ*_2_ = 0 for the normal electric field *E*_z_. The excitation of zero order standing waves, *TM*_0_ and *TE*_0_, are predicted with *k*_*z*_^2^ = *k*_0_^2^*ε*_dielectric_ and *t*_1_ − *t*_0_ = (2*m*π − *φ*_1_ − *φ*_2_)*λ*/(4π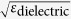)*, m* = 1, 2, 3…, and drawn in Fig. 2 as the white solid lines. For the 1st order *TM*_1_ and *TE*_1_ standing waves, the white dashed lines in Fig. 2 are obtained with *k*_*z*_^2^ = (*ε*_dielectric_ *k*_0_^2^ − *k*_1*,x*_^2^) and *t*_1_ − *t*_0_ *= λp*(2*m*π −* φ*_1_ −* φ*_2_)/(4π
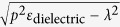
), *m* = 1, 2, 3….. In Fig. 2(a,b), there are three white solid lines corresponding to m = 2, m = 3 and m = 4 from left to right with *φ*_1_ *=* *φ*_2_ = π for *E*_x_; there are also two white dashed lines corresponding to m = 2, m = 3 from left to right with *φ*_1_ *=* *φ*_2_ = π for *E*_x_(also m = 1, m = 2 from left to right with *φ*_1_ *=* *φ*_2_ = 0 for *E*_z_). As shown in Fig. 2(a), deviation between the simulated absorption band and the predicted lines, originated from the PEC assumption of metal, shows a really limited effect on absorption performance. Therefore, the PEC boundary conditions are widely used in this paper.

On the other hand, the excitation wavelength of SPPs in a one-dimensional metal grating can be described by^36^


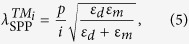


where *ε*_*d*_ is the relative permittivity of the dielectric and *ε*_*m*_ is the real part of the relative permittivity of the metal. Therefore, SPPs supported on the air-Al or dielectric-Al interfaces in our absorber are figured out accordingly, as 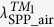 = 6 μm, 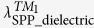 = 9 μm and 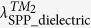 = 4.5 μm. Note that the energy of certain SPPs should be concentrated on the corresponding Floquet mode, e.g., the SPPs energy should be concentrated on the *TM*_1_ mode at 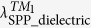, and in the *TM*_2_ mode at 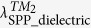. In another words, the excitation of SPPs can also be figured out by the energy distribution analysis of the Floquet modes. This method is crucial for this work, because the twisting of absorption bands causes resonance shifting and Eq. (5) is no longer valid.

**Figure 3:**
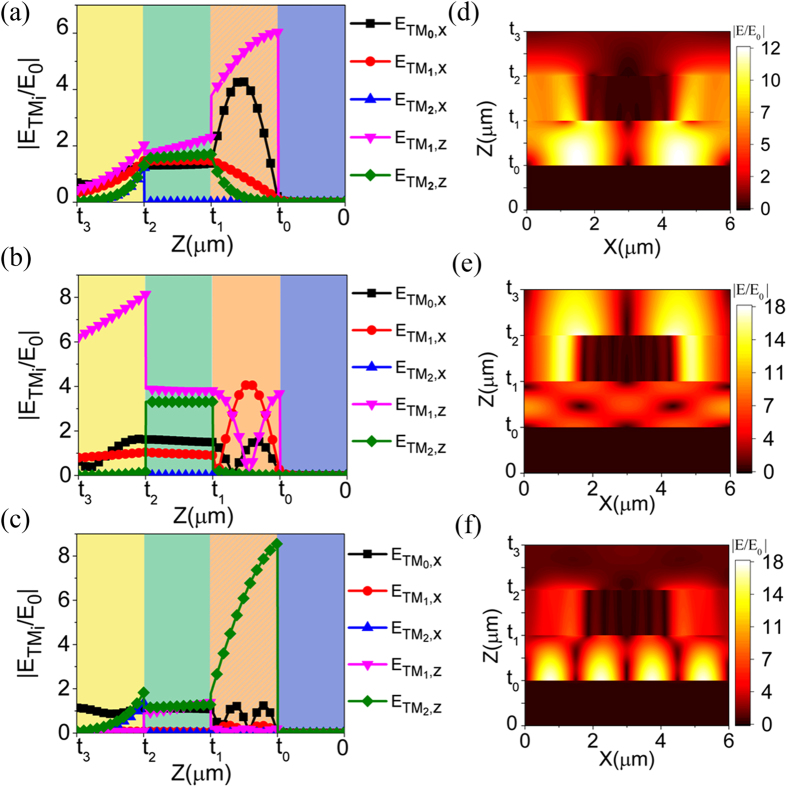
Simulated electric field amplitude distribution of the fundamental modes in the sandwich structured metamaterial absorbers. (**a**–**c**) Modes related E-field distribution along the thickness *Z* direction. (**d**–**f**) Total electric field distribution at the *X*–*Z* cross section. Three cases are carefully chosen from Fig. 2(b) for modes analysis, with (*t*_1_ − *t*_0_, *λ*) of (2.55 μm, 8.65 μm) for (**a**,**d**), (2.85 μm, 6.05 μm) for (**b**,**e**) and (3.45 μm, 4.475 μm) for (**c**,**f**). All the *E*–fields are normalized by the incident electric field *E*_*0*_ in this paper.

To characterize the SPPs related Floquet modes, electric field distribution of three fundamental modes in the proposed metamaterial absorber, *TM*_0_, *TM*_1_ and *TM*_2_, at corresponding absorption peak wavelength are shown in Fig. 3. In Fig. 3(a), the electromagnetic energy mainly distributes on 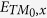 and 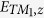, and the studied case is located at the cross region of *TM*_0_ standing waves and *TM*_1_ dielectric-Al SPPs in Fig. 2(b). The energy of *TM*_0_ standing waves could only be in tangential electric field, so it corresponds to 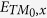. On the other hand, SPPs is a kind of surface wave, so its energy could not be in tangential electric field but correspond to 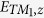. Due to the excitation of standing wave, the energy of 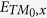 can be enhanced up to 18.5 times the incident energy. The value is calculated by 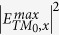, where 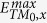 is the maximum of 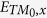 in Fig. 3(a). The *TM*_1_ dielectric-Al SPPs enhances the energy of 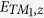 up to 72times the incident energy. The value comes from the maximum of 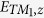 in Fig. 3(a) and then calculated by 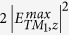. The reason is that the energy is concentrated on the *TM*_1_ mode and its symmetry mode, *TM*_−1_ mode. In Fig. 3(b,e), the dominant *TM*_1_ standing wave and *TM*_1_ air-Al SPPs are evidenced as 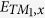 and 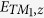 are enhanced in the dielectric layer and on the air-metal interface, respectively. The maximum SPPs energy is about 132 times the incident energy. Unlike the previous two cases, Fig. 3(c,f) just show the *TM*_2_ dielectric-Al SPPs because the enhancement of *E*_*x*_ is not observed, and the energy of SPPs is up to 146 times the incident energy.

## Modes Coupling Analysis

Focusing on the strong absorption nearby the excitation wavelengths of SPPs in Fig. 2(b), it is found that the strong absorption wavelengths can be either larger or smaller than 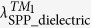 = 9 μm for dielectric-Al SPPs (e.g. the studied case for Fig. 3(a,d)) and keep larger than 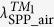 = 6 μm for air-Al SPPs (e.g. the studied case for Fig. 3(b,e)). The reason is that there exists an effective dielectric layer combining the air layer with the dielectric layer. This can be understood by an equivalent approach for the corresponding resonant modes at strong absorption wavelengths.

In the case of dielectric-Al SPPs with 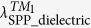 = 9 μm, the *TM*_1_ mode diffraction wave propagates in the dielectric layer with a wavelength of 
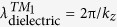
 and *k*_*z*_^2^ = (*ε*_dielectric_*k*_0_^2^ − *k*_1*,x*_^2^). Since the *TM*_1_ mode supports the dielectric-Al SPPs, the enhanced energy mainly concentrates on 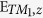. According to Fig. 3(a), 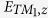 reaches maximum value on the lower dielectric-Al interface and the distribution of 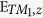 along *Z* direction can be written as 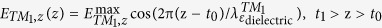. If the thickness of the dielectric layer is smaller than 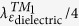, then the enhanced 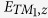 will distribute into the air. Therefore, the SPPs is not simply excited on the lower dielectric-Al interface, but on an equivalent dielectric-metal interface. The equivalent dielectric layer consists of the whole dielectric layer and an air layer with a certain thickness determined by the electric field distribution in the air. As the relative permittivity of air is smaller than *ε*_dielectric_, the equivalent permittivity is smaller than *ε*_dielectric_ as well. Hence, the strong absorption wavelength becomes smaller than 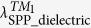 = 9 μm according to Eq. 5. For the same reason, the equivalent dielectric layer should consist the dielectric layer for air-Al SPPs. Since the equivalent relative permittivity is larger than pure air, the wavelengths of strong absorption keep larger than 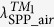 = 6 μm.

**Figure 4:**
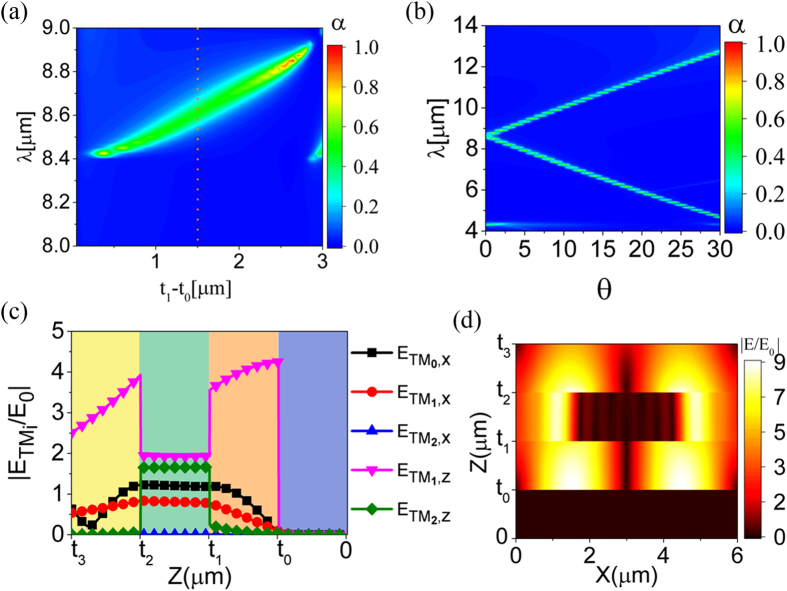
Simulation for the proposed effective dielectric layer in TM polarization. (**a**) Contour plots of the spectral absorption at normal incidence with the air section in Fig. 1(a) replaced by dielectric medium of *ε* = 1.96. (**b**) Contour plots of the spectral absorption as a function of incidence angle *θ* with *t*_1_ − *t*_0_ = 1.5 μm. (**c**,**d**) Modes distribution and total electric field amplitude distribution at (1.5 μm, 8.6 μm) at normal incidence, respectively.

Further evidence for the equivalent approach can be understood by Fig. 4. Since the air section in Fig. 1(a) is replaced by a dielectric medium of *ε* = 1.96, the corresponding excitation wavelength of SPPs on the incident surface moves from 6 μm to 8.4 μm and the strong absorption wavelength slightly increases with the increasing thickness of the dielectric layer (in Fig. 4(a)), consistent with the equivalent approach. In Fig. 4(b), as the incident angle increases, the absorption band splits into two bands, which is the unique character of SPPs. Considering the modes and electric field distribution in Fig. 4(c,d), the 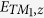 and electric field on both sides of the grating are closely linked. It confirms the origin of strong absorption as SPPs.

**Figure 5:**
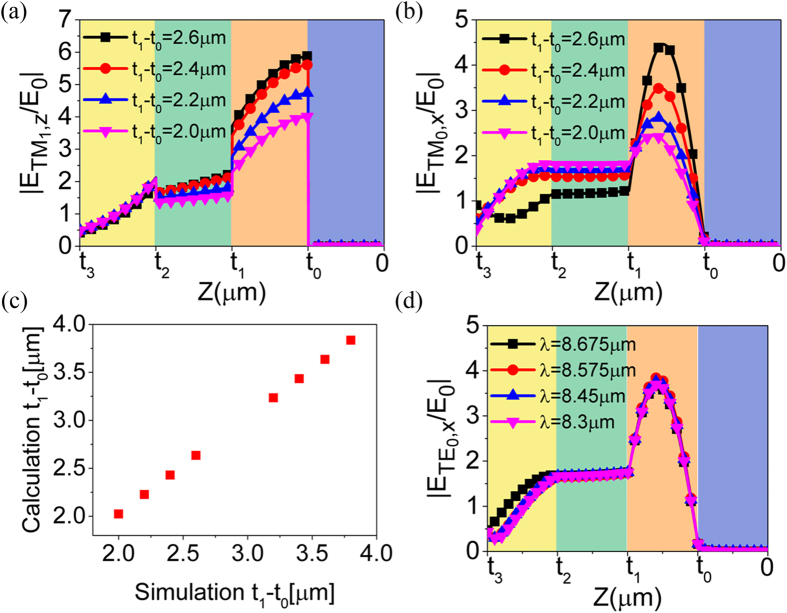
Modes analysis of the coupling between SPPs and standing wave. **(a)** and **(b)** Distribution of 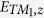 and 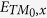 amplitudes at the positions of (2.0 μm, 8.3 μm), (2.2 μm, 8.45 μm), (2.4 μm, 8.575 μm) and (2.6 μm, 8.675 μm) in Fig. 2(b), respectively. **(c)** Comparison of the simulated and calculated thickness of the dielectric layer. **(d)** Distribution of 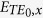 amplitudes at the positions of (2.3 μm, 8.3 μm), (2.35 μm, 8.45 μm), (2.4 μm, 8.575 μm) and (2.45 μm, 8.675 μm) in Fig. 2(a).

Next, we will analyze the coupling between the *TM*_0_ standing wave and the *TM*_1_ dielectric-Al SPPs in the wavelength range of 8.3–8.7 μm in Fig. 2(b). When the thickness of the dielectric layer changes from 2.6 μm to 2.0 μm, the corresponding maximum absorption decreases from 85% to 34% and deviates away from the standing wave line in Fig. 2(b). However, in Fig. 2(a), the corresponding maximum absorption only fells form 60% to 41% and the absorption band is nearly parallel to the standing wave line. The difference is originated from the proposed coupling effect and can be understood by modes characteristics shown in Fig. 5.

In Fig. 5(a,b), when the thickness of the dielectric layer changes from 2.6 μm to 2.0 μm, the coupled *TM*_1_ dielectric-Al SPPs gradually deteriorates as the peak of 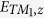 decreases from 5.9 to 4. Meanwhile, the energy transferred from SPPs to the *TM*_0_ standing wave attenuates and causes the reduction of 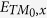 peak from 4.5 to 2.4. In spite of the non-uniform distribution in the resonant dielectric layer, the SPPs strengthened 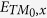 destroys the original continuity of tangential electric field on both sides of the Al grating and forces the electromagnetic energy to redistribute. However, as the Al grating is only 100 nm thick, the tangential electric field on both sides should be continuous to fulfill the boundary condition. In Fig. 5(b), 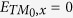 on the bottom dielectric-Al surface. The continuity of tangential electric field can be expressed in the form of


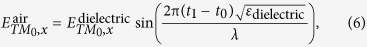


where *t*_1_ − *t*_0_ represents the thickness of dielectric layer and varies, *λ* is the incident wavelength, 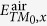 and 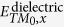 are the amplitude of 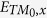 in the air layer and the dielectric layer, respectively. According to Fig. 5(b), the value of 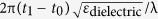 is ranging from π/2 to π. When *t*_1_ − *t*_0_ changes from 2.6 μm to 2.0 μm, 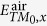 increases and 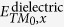 decreases. In this case, 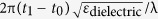 should be decreased to fulfill the boundary condition. In another words, λ/(*t*_1_ − *t*_0_) should be increased. Since the standing wave line has a constant λ/(*t*_1_ − *t*_0_), the absorption band must gradually deviate from the standing wave line, as is shown in Fig. 2(b). Figure 5(c) is the comparison of simulation and calculation results about the thickness of the dielectric layer and they are very close. The calculation results are get by picking up a series of 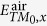
and 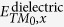 from Fig. 5(b) and then computing by Eq. 6. Therefore, the analysis approach based on boundary condition is confirmed quantitatively.

In Fig. 5(d), there is almost no change of 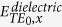 for TE polarization when incident wavelength varies as the same way for TM polarization. It means that the standing waves for TE polarization are very stable without the coupling to SPPs. By comparing Fig. 5(b) with 5(d), it is found that the coupling affects standing waves in two ways: one is the reduction of 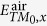 caused by absorption enhancement of SPPs; the other is the enhancement of 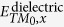 caused by the energy transferred from SPPs, when the incident wavelength is close to 9 μm. These two opposite rules of variation and the continuity of tangential electric field mean that there is only a certain incident wavelength corresponding to a certain thickness of dielectric layer to excite the coupled SPPs efficiently. Thus, the excitation wavelength of the coupled SPPs is not exactly at 9 μm but around 9 μm when the thickness of dielectric layer varies around 3 μm. Therefore, the absorption bands at the cross region of SPPs and standing waves are twisted in Fig. 2(b,c).

## Conclusion

In summary, a comprehensive analysis about the sandwich structure with different thickness of the dielectric layer is conducted in this paper. The relationships between modes distribution and coupling are figured out by RCWA method. Experimental results of absorption spectrum support the validity of our simulations. The shift of excitation wavelength of SPPs is explained by the effective dielectric method and the coupling between SPPs and standing waves is dedicatedly studied through the distribution of different modes. The coupling between SPPs and standing waves shows a new way to study and manipulate SPPs and a method to achieve the transfer of energy between different modes. A new approach based on the boundary conditions is successfully introduced to reveal the coupling mechanism and the corresponding frequency shifting phenomenon. This study will assist in improving the detection sensitivity of sensors and emissivity efficiency of infrared emitters.

## Methods

Numerical simulations are performed by RCWA method. The proposed sandwich structure absorber is prepared by standard e-beam deposition and UV lithography techniques. The 200 nm-thick Al ground plane was evaporated onto a silicon substrate and then covered by the Al_2_O_3_ layer with different thickness. Well patterning of the 100 nm-thick Al film was realized by the lift-off lithography process on Al_2_O_3_ layer. The experimental results are tested by PerkinElmer FT-IR Microscope.

## Additional Information

**How to cite this article**: Zhen, G. *et al*. Modes Coupling Analysis of Surface Plasmon Polaritons Based Resonance Manipulation in Infrared Metamaterial Absorber. *Sci. Rep.* **7**, 46093; doi: 10.1038/srep46093 (2017).

**Publisher's note:** Springer Nature remains neutral with regard to jurisdictional claims in published maps and institutional affiliations.

## Supplementary Material

Supplementary Information

## References

[b1] Murray W. A. & Barnes W. L. Plasmonic materials. Adv. Mater. 19, 3771–3782 (2007).

[b2] Pitarke J. M., Silkin V. M., Chulkov E. V. & Echenique P. M. Theory of surface plasmons and surface-plasmon polaritons. Rep. Prog. Phys. 70, 1–87 (2007).

[b3] García-Vidal F. J., Moreno E., Porto J. A. & Martín-Moreno L. Transmission of light through a single rectangular hole. Phys. Rev. Lett. 95, 103901 (1999). 10.1103/PhysRevLett.95.103901 16196929

[b4] Gramotnev D. K. & Bozhevolnyi S. I. Plasmonics beyond the diffraction limit. Nat. Photonics. 4, 83–91 (2004).

[b5] Anker J. N. . Biosensing with plasmonic nanosensors. Nature Mater. 7, 442–453 (2008). 18497851 10.1038/nmat2162

[b6] Rahul D., Vladislav J., Saeed H. & Dietmar K. Analyzing periodic and random textured silicon thin film solar cells by rigorous coupled wave analysis. Sci. Rep. 4, 6029 (2014). 25112301 10.1038/srep06029 PMC5381427

[b7] Wang L. P. & Zhang Z. M. Wavelength-selective and diffuse emitter enhanced by magnetic polaritons for thermophotovoltaics. Appl. Phys. Lett. 100, 063902 (2012).

[b8] Carrillo S. G. . Design of practicable phase-change metadevices for near-infrared absorber and modulator applications. Opt. Express 24, 13563 (2016). 27410372 10.1364/OE.24.013563

[b9] Lee B. J., Wang L. P. & Zhang Z. M. Coherent thermal emission by excitation of magnetic polaritons between periodic strips and a metallic film. Opt. Express 16, 11328–36 (2008). 18648451 10.1364/oe.16.011328

[b10] Wang L. P. & Zhang Z. M. Effect of magnetic polaritons on the radiative properties of double-layer nanoslit arrays. J. Opt. Soc. Am. B 27, 2595–2604 (2010).

[b11] Cheng C. W. . Wide-angle polarization independent infrared broadband absorbers based on metallic multi-sized disk arrays. Opt. Express 20, 10376–81 (2012). 22535127 10.1364/OE.20.010376

[b12] Chen Y. G. . Hybrid phase-change plasmonic crystals for active tuning of lattice resonances. Opt. Express 21, 13691 (2013). 23736622 10.1364/OE.21.013691

[b13] Wang H. & Wang L. P. Perfect selective metamaterial solar absorbers. Opt. Express 21, A1078–93 (2013). 24514927 10.1364/OE.21.0A1078

[b14] Chen K., Adato R. & Altug H. Dual-band perfect absorber for multispectral plasmon-enhanced infrared spectroscopy. ACS Nano 6, 7998–8006 (2012). 22920565 10.1021/nn3026468

[b15] Yan M., Dai J. & Qiu M. Lithography-free broadband visible light absorber based on a mono-layer of gold nanoparticles. J. Optics 16, 025002 (2014).

[b16] Nath J. . Infra-red spectral microscopy of standing-wave resonances in single metal-dielectric-metal thin-film cavity. Proc. of SPIE 9544, 95442M(2015).

[b17] Zhang N. . Broadband absorption in mid-infrared metamaterial absorbers with multiple dielectric layers. Opt. Commun. 338, 388–392 (2015).

[b18] Zhang N. . Dual-band absorption of mid-infrared metamaterial absorber based on distinct dielectric spacing layers. Opt. Lett. 38, 1127 (2013). 10.1364/OL.38.001125 23546265

[b19] Ruan Z. C. & Qiu M. Enhanced transmission through periodic arrays of subwavelength holes: the role of localized waveguide resonances. Phys. Rev. Lett. 96, 233901 (2006). 16803379 10.1103/PhysRevLett.96.233901

[b20] Yun H., Lee S. Y. & Lee B. Hybrid Multibands of Surface Plasmon and Fabry–Pérot Resonances. IEEE Photonics Techl. 26, 2027–30 (2014).

[b21] Zhang S., Genov D. A., Wang Y., Liu M. & Zhang X. Plasmon-induced transparency in metamaterials. Phys. Rev. Lett. 101, 074401 (2008). 10.1103/PhysRevLett.101.047401 18764363

[b22] Halaev V. M. . Negative index of refraction in optical metamaterials. Opt. Lett. 30, 3356–8 (2005). 16389830 10.1364/ol.30.003356

[b23] Chen J. X., Wang P., Lu Y. H. & Ming H. Coupling between gap plasmon polariton and magnetic polariton in a metallic-dielectric multilayer structure. Phys. Rev. E 84, 026603 (2011). 10.1103/PhysRevE.84.026603 21929124

[b24] Wang H. & Wang L. P. Tailoring thermal radiative properties with film-coupled concave grating metamaterials. J. Quant. Spectrosc. R. A. 158, 127–135 (2015).

[b25] Liu N., Mesch M., Weiss T., Hentschel M. & Giessen H. Infrared perfect absorber and its application as plasmonic sensor. Nano Lett. 10, 2342–8 (2010). 20560590 10.1021/nl9041033

[b26] Zhang Z. J., Park K. & Lee B. J. Surface and magnetic polaritons on two-dimensional nanoslab-aligned multilayer structure. Opt. Express 19, 16375–89 (2012). 10.1364/OE.19.016375 21935001

[b27] Nath J. . Far-infrared absorber based on standing-wave resonances in metal-dielectric-metal cavity. Opt. Express 23, 20366–80 (2015). 26367892 10.1364/OE.23.020366

[b28] Peng X. Y., Wang B., Lai S., Zhang D. H. & Teng J. H. Ultrathin multi-band planar metamaterial absorber based on standing wave resonances. Opt. Express 20, 27756–60 (2012). 23262721 10.1364/OE.20.027756

[b29] Collin R. E. Field Theory of Guided Waves, IEEE Press (1991).

[b30] Lacasse J. D. & Laurin J. J. A Method for Reflectarray Antenna Design Assisted byNear Field Measurements. IEEE Trans. Antennas Propag. 54, 1891 (2006)

[b31] Mittra. R., Chan C. H. & Cwik T. Techniques for Analyzing Frequency Selective Surfaces-A Review. P. IEEE 76, 1593 (1988)

[b32] Ordal M. A. . Optical properties of the metals Al, Co, Cu, Au, Fe, Pb, Ni, Pd, Pt, Ag, Ti, and W in the infrared and far infrared. Appl. Optics 22, 1099–20 (1983). 10.1364/ao.22.001099 18195926

[b33] Kischkat J. . Mid-infrared optical properties of thin films of aluminum oxide, titanium dioxide, silicon dioxide, aluminum nitride, and silicon nitride. Appl. Optics 51, 6789–98 (2012). 10.1364/AO.51.006789 23033094

[b34] Li Z. Y. & Lin L. L. Photonic band structures solved by a plane-wave-based transfer-matrix method. Phys. Rev. E, 67, 046607 (2003). 10.1103/PhysRevE.67.046607 12786509

[b35] Li L. F., Cindrich I. & Lee S. H. New formulation of the Fourier modal method for crossed surface-relief gratings. J. Opt. Soc. Am. A 14, 2758–67 (1997).

[b36] Barnes W. L. Surface plasmon–polariton length scales:a route to sub-wavelength optics. J. Opt. A-Pure Appl. O. P. 8, S87–93 (2006).

