# Supplementary material for: Modes Coupling Analysis of Surface Plasmon Polaritons Based Resonance Manipulation in Infrared Metamaterial Absorber
Source: Sci Rep. 2017 Apr 11;7:46093. doi: 10.1038/srep46093 (PMC5387402; doi:10.1038/srep46093)
Supplement: Supplementary Information [file srep46093-s1.pdf]

Supporting Information

Modes Coupling Analysis of Surface Plasmon Polaritons Based Resonance Manipulation in Infrared Metamaterial Absorber

Guoshuai Zhen, Peiheng Zhou, XiaojiaLuo, Jianliang Xie and Longjiang Deng
National Engineering Research Center of Electromagnetic Radiation Control Materials,
State Key Laboratory of Electronic Thin Film and Integrated Devices,
University of Electronic Science and Technology of China,
Chengdu 610054, Sichuan, People's Republic of China

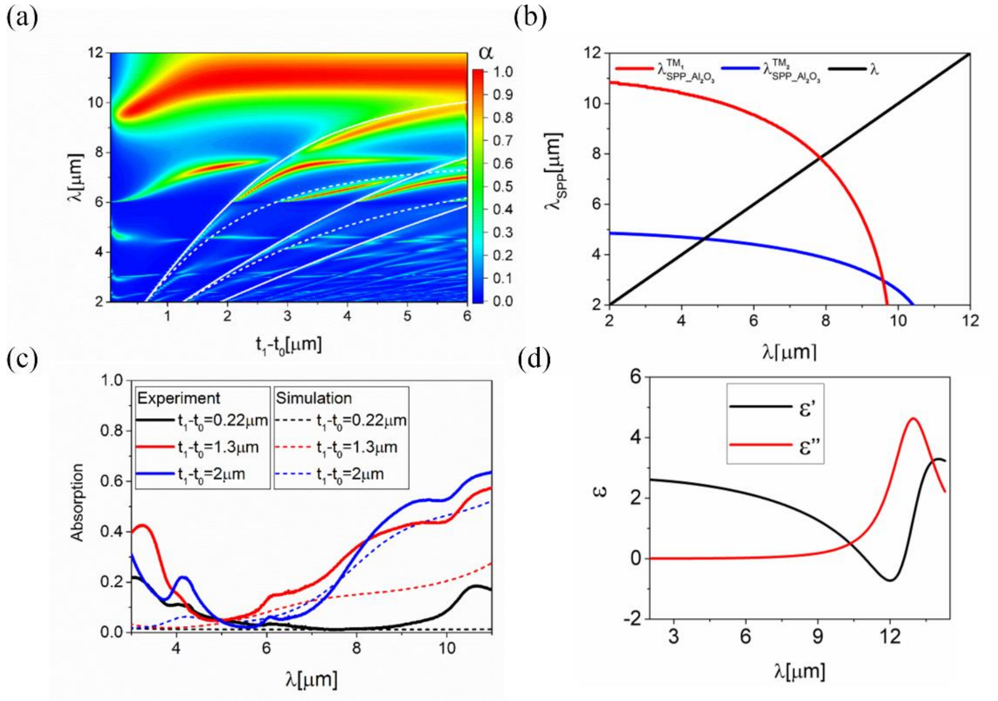

Figure S1. (Color online) Simulation and experimental results of the sandwich structured metamaterial absorber at normal incidence. (a) Contour plots of the spectral absorption α as a function of incident wavelength λ and thickness of the Al_2O_3 layer for TM polarization. The white solid lines represent the predicted excitation of the zero order standing wave and the white dashed lines are for the first order one. **(b)** Calculation of the excitation wavelengths of TM_1 and TM_2 Al_2O_3-Al SPPs based on the dispersion of Al_2O_3 shown in [1]. **(c)** Comparison of the simulation and experimental absorption without the grating. Three absorber samples selected at the characteristic regions of Fig. S1(a) are defined by the thickness of the Al_2O_3 layer, with t_1-t_0=0.22μm, 1.3μm and 2μm. **(d)** The relative permittivity of Al_2O_3 get from [1].

Experimental Results. The proposed sandwich structure absorber is prepared by standard e-beam deposition and UV lithography techniques. The 200nm-thick Al ground plane was evaporated onto a silicon substrate and then covered by the Al_2O_3

layer with different thickness. Lamellar grating of the 100nm-thick Al film was realized by the lift-off lithography process on Al_2O_3 layer.

Fig. S1(a) keeps the same absorption pattern as it is shown in Fig. 2(b) in our article. One of the main differences is that the TM_0 standing waves lines are no longer straight lines because of the dispersion of Al_2O_3, as the white solid lines shows in Fig. S1(a). In addition, the excitation wavelengths of Al_2O_3-Al SPPs are calculated by

$$\lambda_{\mathrm{SPP}}^{TM_i}=\frac{p}{i}\sqrt{\frac{\varepsilon_d\varepsilon_m}{\varepsilon_d+\varepsilon_m}}, \tag{1}$$

where ε_d is the relative permittivity of the dielectric, ε_m is the real part of the relative permittivity of the metal, p is the period of grating and i represents the Flouqet modes. Because Al_2O_3 is dispersive, the excitation wavelengths of TM_1 and TM_2 Al_2O_3-Al SPPs can't be calculated directly, but they could be solved graphically like Fig. S1(b). The calculation results of $\lambda_{\mathrm{SPP\_Al_2O_3}}^{TM_1}$=7.83μm and $\lambda_{\mathrm{SPP\_Al_2O_3}}^{TM_2}$=4.62μm match well with the simulation results of $\lambda_{\mathrm{SPP\_Al_2O_3}}^{TM_1}$=7.8μm and $\lambda_{\mathrm{SPP\_Al_2O_3}}^{TM_2}$=4.58μm obtained from Fig. S1(a). Another main difference is the strong absorption at the top of Fig. S1(a) when the incident wavelength is larger than 10μm and it mixes together with the strong absorption caused by magnetic polaritons. Considering the experimental results in Fig. S1(c), two wavelength regions with strong absorption are around λ=3μm and λ= 10μm, respectively. So the absorption at the top of Fig. S1(a) is caused by the intrinsic dielectric loss of Al_2O_3. There is another absorption peak at λ=4.13μm with t_1-t_0=2μm both in simulation and experimental results in Fig. S1(c). Considering the fact that the thickness of Al_2O_3 layer is just 3/4 of the effective wavelength, the absorption peak is caused by standing waves. However, for the absorption peak at λ=3.24μm, the thickness of Al_2O_3 layer is far lower than 3/4 of the effective wavelength and a thinner Al_2O_3 layer causes stronger absorption, so it is not caused by standing waves but the intrinsic dielectric loss of Al_2O_3.

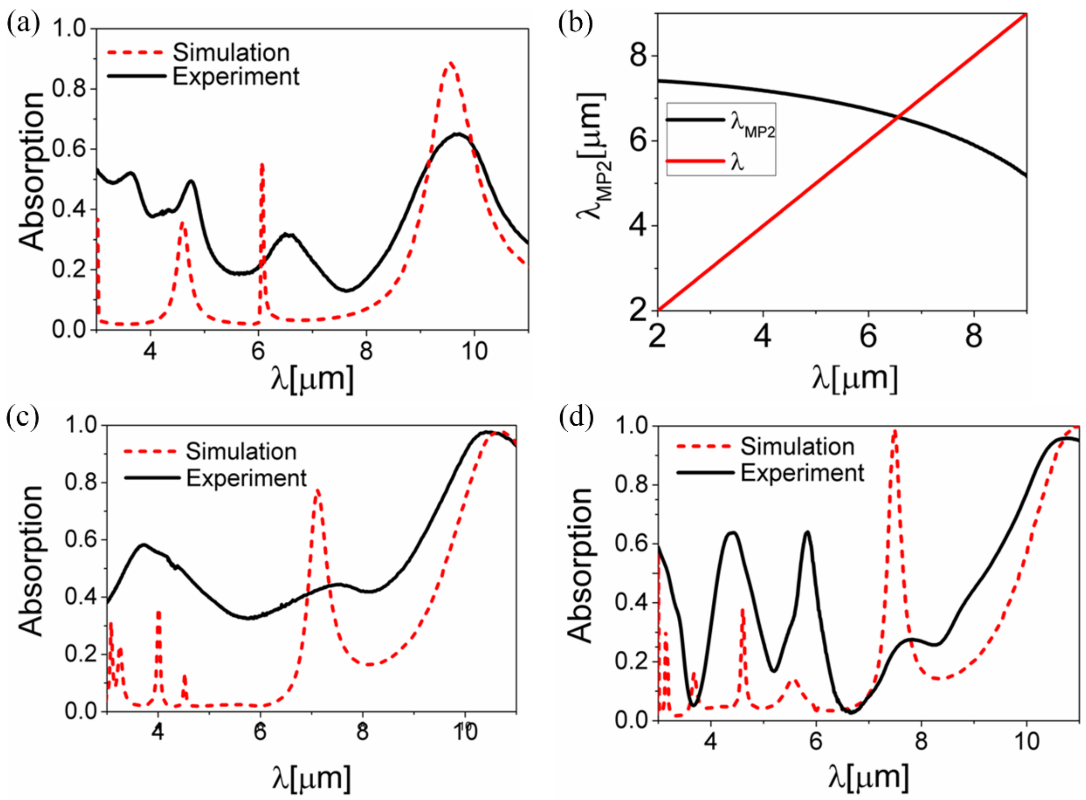

Figure S2. (**a, c, d**) Simulation and experimental results of three samples with t_1-t_0=0.22μm, 1.3μm and 2μm at normal incidence, respectively. **(b)** Calculation of the excitation wavelength of MP_2 based on the result of MP_3 and the dispersion of Al_2O_3 shown in R[1].

In Fig. S2(a), the absorption peaks of experimental results at λ=9.54μm, 6.49μm, 4.75μm, 3.62μm and 3μm are caused by magnetic polariton MP_1, MP_2 mixing TM_1 air-metal SPPs, MP_3, MP_4 and the intrinsic dielectric loss of Al_2O_3, respectively. The multiple magnetic polariton branches, denoted as MP_1, MP_2, MP_3 and MP_4, correspond to the fundamental, second, third and fourth harmonic resonances, respectively. The frequency of MP_1 can be calculated by f_r=c/2π$d\sqrt{\varepsilon_d c_1/2}$ [2] and the frequency of MP_i is around the i times of MP_1 for non-dispersive dielectric and i=1,2,3.... [4]. However, for dispersive dielectrics, like Al_2O_3, the wavelength of MP_1 is much smaller than three times the wavelength of MP_3. Because the relative permittivity of Al_2O_3 at λ=9.54μm is 1.01, which is much smaller than 2.36 at λ=4.75μm. If the change of relative permittivity is considered and c_1 keeps constant, the wavelength of MP_2 should be at λ=4.86μm, which is very close to λ=4.75μm. The absorption peak at λ=3.62μm is not observed in simulation but it may be caused by MP_4, which could not be observed at normal incidence [3-4]. The relative permittivity of Al_2O_3 at λ=3.62μm is 2.49, which is close to 2.36 at λ=4.75μm. And the wavelength of MP_4 (λ=3.62μm) is 3/4 of the wavelength of MP_3 (λ=4.75μm). In Fig, S2(b), the wavelength of MP_2 is calculated based on the wavelength of MP_3. The wavelength of MP_2 is at λ=6.56μm and close to the excitation wavelength of TM_1 air-metal SPPs at 6.06μm in simulation, so we think that MP2 and TM_1 air-metal SPPs are excited together at λ=6.49μm.

In Fig, S2(c), the absorption peaks of experimental results at λ=10.4μm, 7.525μm and 3.775μm are caused by the intrinsic dielectric loss of Al_2O_3, TM_1 effective dielectric-metal SPPs and TM_0 standing waves, respectively. In Fig, S2(d), the

absorption peaks of experimental results at λ=10.75μm, 7.82μm, 5.84μm, 4.41μm and 3μm are caused by the intrinsic dielectric loss of Al_2O_3, TM_1 effective dielectric-metal SPPs, TM_0 standing waves, TM_2 Al_2O_3-metal SPPs and the intrinsic dielectric loss of Al_2O_3, respectively. Here are obvious differences between simulation and experimental results in Fig. S2(c) and S2(d), the main reason is that the growth of Al_2O_3 layer by e-beam deposition is not well when the thickness of Al_2O_3 layer is larger than 1μm. The Al_2O_3 layer peels off from the substrate in many cases and for the successfully deposited samples there are still many cracks on the surface.

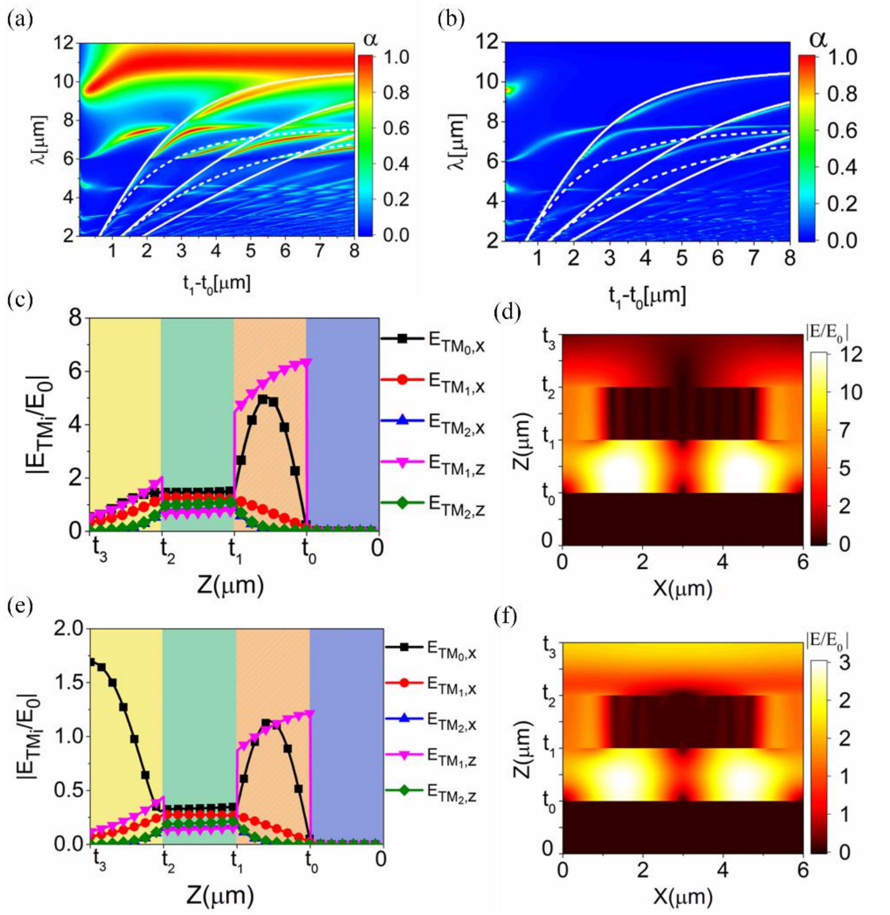

Figure S3. The comparison of modes distribution and total electric field distribution with lossy and lossless Al_2O_3 layer (with the dispersive real part of the permittivity). (**a, b**) Contour plots of the spectral absorption α with lossy and lossless Al_2O_3 layer, respectively. **(c, d)** Modes distribution and total electric field distribution with lossless Al_2O_3 layer. **(e, f)** Modes distribution and total electric field distribution with lossy Al_2O_3 layer. The same position is selected at the Al_2O_3 layer thickness and incident wavelength of (2.6μm, 7.68μm) in Fig. S3(a) and S3(b).

Effects of the dielectric loss. The lossless Al_2O_3 ignores the imaginary part of the relative permittivity of Al_2O_3, so the absorption caused by the intrinsic dielectric loss of Al_2O_3 is eliminated at the top of Fig. S3(b). However, the absorption caused by the sandwich structure keep at the same position in both Fig. S3(a) and S3(b). Due to the dielectric loss of Al_2O_3, the value of absorption is changed. However, the quantitative analysis of this change is beyond the scope of our theory. Our theory focuses on the coupling between SPPs and standing waves and it handles well with the balance

between SPPs and standing waves. When the dielectric loss of Al_2O_3 is considered, the balance is among SPPs, standing waves and the dielectric loss of Al_2O_3 and it becomes more complicated.

The position (2.6μm, 7.68μm) is selected in Fig. S3(a) and S3(b) to analyze the effects of dielectric loss. Comparing with Fig. S3(c), even though the $E_{TM_0,x}$ and $E_{TM_1,z}$ in the Al_2O_3 layer keep the same distribution and the same relative amplitude in Fig. S3(e), the absolute value of them are sharply weakened by the dielectric loss. Meanwhile, the total electric field in the Al_2O_3 layer still keeps the same distribution in Fig. S3(f) but the maximum is only a quarter of the maximum in Fig. S3(d). The absorption with lossless Al_2O_3 layer is 0.7. Due to the strong suppression of standing wave and SPPs by dielectric loss, the absorption with lossy Al_2O_3 layer is only 0.52. At position (2.6μm, 7.68μm), the dominant absorption mechanism is the coupling between SPPs and standing waves. The dielectric loss has negative effects on the absorption.

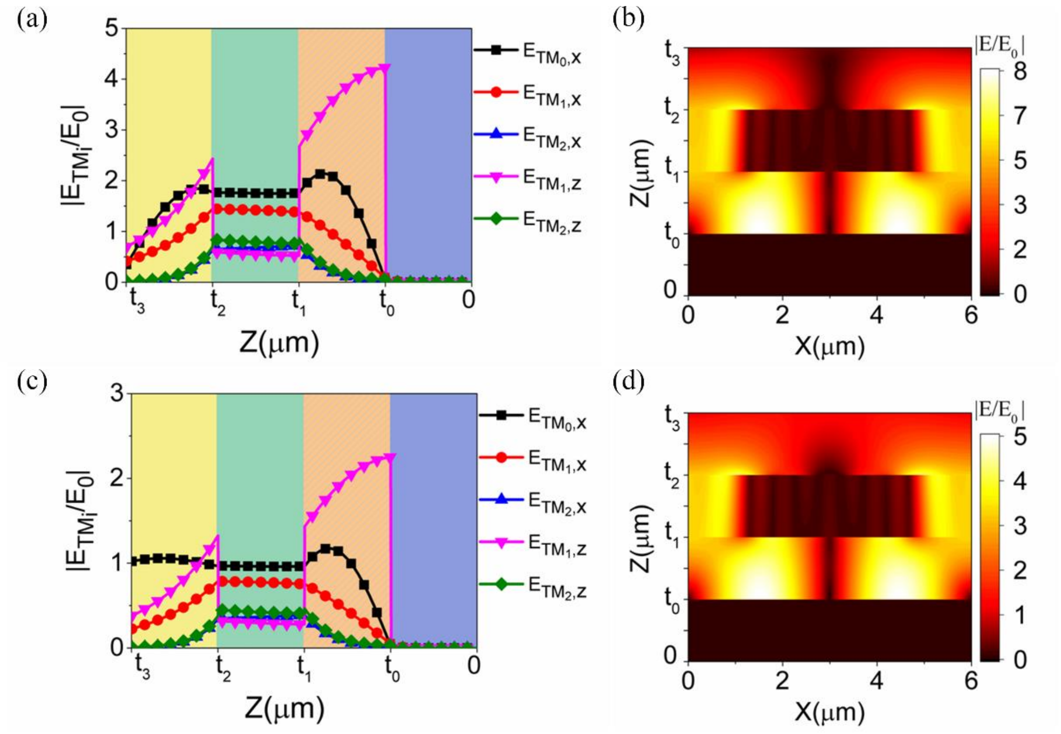

Figure S4. The comparison of modes distribution and total electric field distribution with lossy and lossless Al_2O_3 layer. (a, b) Modes distribution and total electric field distribution with lossless Al_2O_3 layer. **(c, d)** Modes distribution and total electric field distribution with lossy Al_2O_3 layer. The same position is selected at the Al_2O_3 layer thickness and incident wavelength of (1.9μm, 7.46μm) in Fig. S3(a) and S3(b).

The position (1.9μm, 7.46μm) is an opposite case and the dielectric loss has positive effects on the absorption. The absorption with lossless Al_2O_3 layer is 0.29 and the absorption with lossy Al_2O_3 layer is 0.99. Even though there is the suppression of standing wave and SPPs by dielectric loss as well, the suppression at (1.9μm, 7.46μm) is far lower than the suppression at (2.6μm, 7.68μm). In the Al_2O_3 layer, the $E_{TM_0,x}$ and $E_{TM_1,z}$ in Fig. S4(c) is nearly half of that in Fig. S4(a), and the maximum of total electric field in Fig. S4(d) is more than half of that in Fig, S4(b). Because the lossy Al_2O_3 layer retains strong electric field distribution as well, the dominant absorption mechanism is

the dielectric loss of Al_2O_3 at position (1.9μm, 7.46μm).

Based on the above analysis, it could be find that the dielectric loss has effects on the energy of Floquet modes, but it can't change the distribution of Floquet modes in the dielectric layer. The dielectric loss has an effect on the value of absorption but has no effect on the absorption peak position. So the analysis of sandwich structure with lossy dielectric can base on the results with the corresponding lossless dielectric and the qualitative analysis as described above. This is a simple and effective method to handle the balance among SPPs, standing waves and the dielectric loss of Al_2O_3.

1. Kischkat, J. et al. Mid-infrared optical properties of thin films of aluminum oxide, titanium dioxide, silicon dioxide, aluminum nitride, and silicon nitride. *Appl. Optics* **51**, 6789-98 (2012).

2. Zhou, J., Economon, E. N., Koschny, T. & Soukoulis, C. M. Unifying approach to left-handed material design. *Opt. Letters* **31**, 3620-2 (2006).

3 .Lee, B. J., Wang, L. P. & Zhang, Z. M. Coherent thermal emission by excitation of magnetic polaritons between periodic strips and a metallic film. *Opt. Express* **16**, 11328-36 (2008).

4. Zhang, Z. J, Park, K. & Lee, B. J. Surface and magnetic polaritons on two-dimensional nanoslab-aligned multilayer structure. *Opt. Express* **19**, 16375-89 (2011).
